# Development and internal validation of a machine learning-based disease burden index for irritable bowel syndrome: a multicentre cross-sectional study

**DOI:** 10.1080/07853890.2026.2721087

**Published:** 2026-08-20

**Authors:** Yanbin Wei, Heyang Zhang, Nan Wang, Haifeng Jin, Zitan Feng, Jiapeng Guo, Jiafei Peng, Jia Feng, Jia Zhi, Shutian Zhang, Shengtao Zhu, Xin Yao

**Affiliations:** ^a^ Department of Gastroenterology, Beijing Friendship Hospital, Capital Medical University, Beijing, P. R. China; ^b^ State Key Laboratory of Digestive Health, Beijing, P. R. China; ^c^ National Clinical Research Center for Digestive Disease, Beijing, P. R. China; ^d^ Beijing Key Laboratory of Early Gastrointestinal Cancer Medicine and Medical Devices, Beijing, P. R. China; ^e^ Department of Gastroenterology, Bethune International Peace Hospital, Shijiazhuang, Hebei, P. R. China; ^f^ School of Clinical Medicine, Chengdu University of Traditional Chinese Medicine, Chengdu, Sichuan, P. R. China

**Keywords:** Irritable Bowel Syndrome, Rome Criteria, Latent Class Analysis, Machine Learning, Disease Burden

## Abstract

**Background:**

Irritable bowel syndrome (IBS) is one of the most common disorders of gut-brain interaction in gastroenterology clinics worldwide, becoming a significant public health burden. This study aims to provide an exploratory approach for comprehensive assessment of IBS burden and may serve as a basis for future external validation and further evaluation of its potential clinical application.

**Patients and Methods:**

This multicentre cross-sectional study included IBS patients diagnosed according to the Rome III and/or Rome IV criteria. Rome criteria-based comparisons and latent class analysis (LCA) were performed to characterize clinical heterogeneity, followed by the development of the IBS burden index (IBS-BI) using machine learning.

**Results:**

Compared to the Rome IV-ineligible group, the Rome IV-eligible group exhibited significantly more severe IBS symptoms, somatization symptoms, anxiety symptoms, depression symptoms, and poorer health-related quality of life (all *p* <0.05). Linear regression analysis showed that the severity of these symptoms was inversely related to health-related quality of life (all *p* <0.05). LCA identified four heterogeneous IBS burden phenotypes: high-symptom burden, low-symptom burden, somatization-dominant, and psychological comorbid phenotypes. Using machine learning, we developed the IBS-BI and an interactive clinical calculator. The validation was limited to an internal training-validation split, and no external validation was performed.

**Conclusions:**

Patients with IBS who fulfilled the Rome IV criteria showed a higher disease burden, which may be related to the more stringent diagnostic requirements of these criteria. The IBS-BI may have potential value for evaluating disease burden and patient stratification. Its clinical utility and broader applicability require further validation in independent external cohorts and prospective studies.

## Introduction

Irritable bowel syndrome (IBS) is a functional gastrointestinal disorder characterized by recurrent abdominal pain, accompanied by changes in bowel habits and stool characteristics. Its development involves complex interactions among impaired gut-brain communication, visceral hypersensitivity, gut microbiota dysbiosis, and psychological factors, making it one of the most common disorders of gut-brain interaction worldwide [1]. A systematic review and meta-analysis of epidemiological studies estimated the global prevalence of IBS to be approximately 15.0%, with a gradually increasing trend in recent years [2]. However, considerable heterogeneity remains in the reported prevalence of IBS due to variations in diagnostic criteria, study designs, and geographical regions [3,4]. Although IBS is not a life-threatening condition, its chronic and recurrent nature, together with its multifactorial pathogenesis, imposes a substantial physical and psychological burden on patients. IBS significantly impairs quality of life and daily functioning, while also increasing healthcare utilization and socioeconomic costs, representing an important global public health challenge [5,6].

IBS subtypes were initially proposed within the framework of the Rome III criteria based on the Bristol Stool Form Scale. Patients were classified into four major subtypes: IBS with predominant constipation (IBS-C), IBS with predominant diarrhoea (IBS-D), IBS with mixed bowel habits (IBS-M), and IBS unclassified (IBS-U) [7]. Although the Rome IV criteria, introduced in 2016, incorporated updated insights into biopsychosocial factors and gut-brain interactions, the classification system remains primarily based on gastrointestinal symptoms and does not include important dimensions such as somatization, anxiety, depression, or quality of life [8]. Therefore, some studies have attempted to move beyond traditional IBS subtypes by incorporating psychological burden and quality-of-life measures to identify more clinically meaningful patient phenotypes. Several studies have classified IBS patients into 4–7 distinct clusters, providing new perspectives for personalized management [9,10]. However, differences in study populations, data sources, and assessment approaches have resulted in inconsistent findings, and these classification systems have not yet been integrated into a quantitative framework for assessing overall disease burden, limiting their clinical utility.

In addition, commonly used IBS assessment tools mainly focus on individual domains, such as gastrointestinal symptoms, psychological status, or quality of life [11–13]. Although multiple assessment tools can provide information across different clinical domains, interpreting these measures separately may increase complexity and may not adequately reflect the overall disease burden of IBS. Therefore, an integrated tool combining gastrointestinal symptoms, psychological status, and quality of life is needed. Such a tool can consolidate multidimensional clinical information into a single measure, facilitating IBS burden quantification, patient stratification, individualized management, treatment response evaluation, and long-term monitoring.

This study conducted a cross-sectional analysis based on the demographic characteristics of IBS patients, incorporating standardized scale assessments of IBS symptoms, somatization symptoms, anxiety symptoms, depression symptoms, and health-related quality of life. We systematically compared the multidimensional clinical characteristics of IBS patients who met and did not meet the Rome IV criteria. Subsequently, latent class analysis (LCA) was performed to identify homogeneous clinical phenotypes of IBS patients. Based on these findings, we developed the IBS Burden Index (IBS-BI) to integrate multidimensional clinical information and provide a quantitative measure of overall disease burden. A simplified clinical tool was also developed to facilitate IBS-BI calculation. This study aims to provide an exploratory approach for comprehensive assessment of IBS burden, and may serve as a basis for future external validation and further evaluation of its potential clinical application.

## Patients and methods

### Study design and Ethics

This multicentre cross-sectional study was conducted in the Bethune International Peace Hospital (July 2017 to September 2018) and Beijing Friendship Hospital, Capital Medical University (September 2021 to March 2026). The study protocol was approved by the Ethics Committee of Bethune International Peace Hospital (2016-KY-020) and Beijing Friendship Hospital, Capital Medical University (2021-P2-239-01). Identical standardized diagnostic procedures, questionnaires, interviewer training, and data collection protocols were applied at both centers to ensure consistency in study procedures and data comparability. All participants underwent simultaneous assessment based on Rome III and Rome IV diagnostic criteria on the day of enrollment. The study was conducted in accordance with the Strengthening the Reporting of Observational Studies in Epidemiology statement (Supplementary File 1). All participants were rigorously selected according to inclusion and exclusion criteria.

### Study population

**Figure 1.**
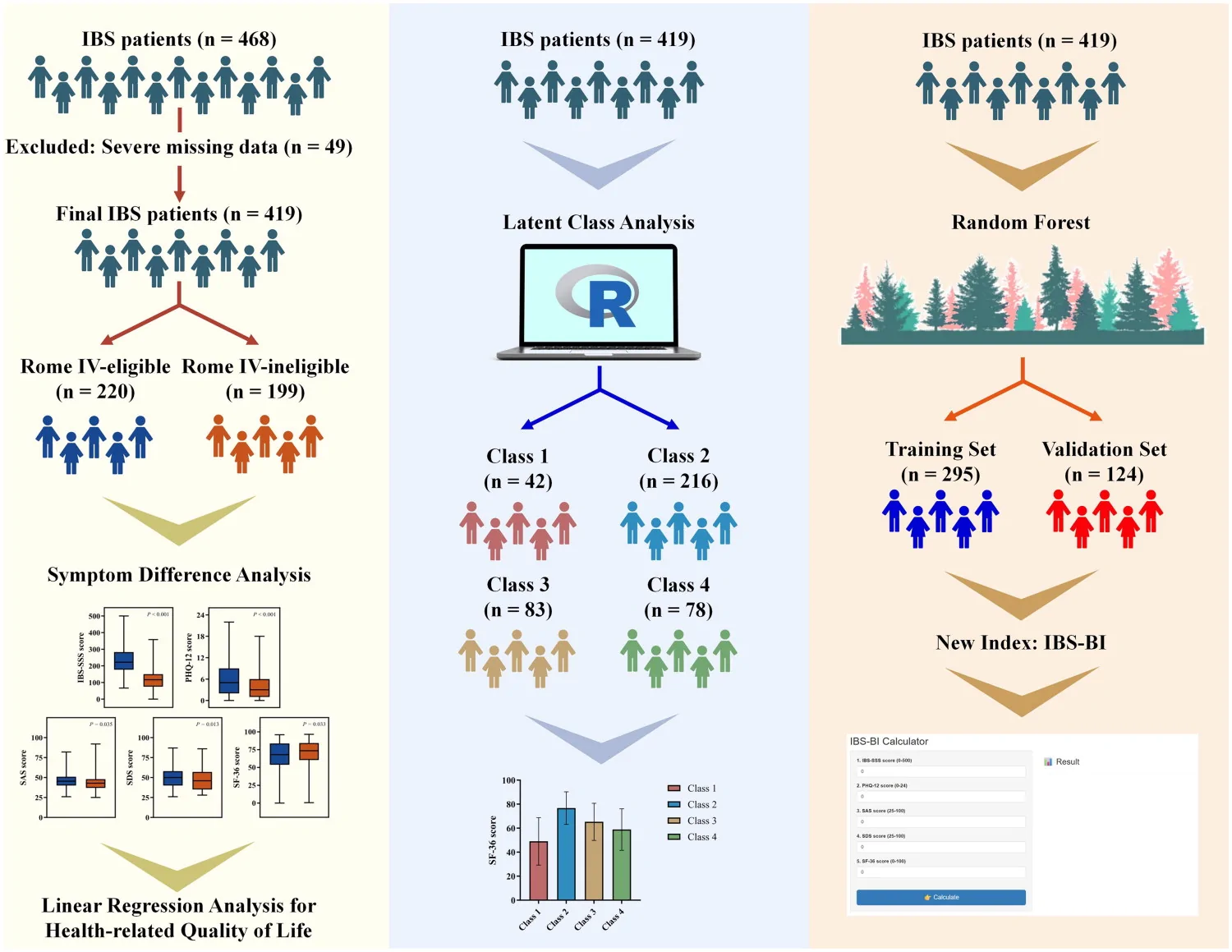
Study inclusion, exclusion criteria and research framework. IBS, irritable bowel syndrome; IBS-BI, IBS Burden Index; IBS-SSS, IBS-Severity Scoring System; PHQ-12, Patient Health Questionnaire-12; SAS, Self-Rating Anxiety Scale; SDS, Self-Rating Depression Scale; SF-36, 36-Item Short Form Health Survey.

A total of 468 patients aged 18–75 years who met the Rome III and/or Rome IV criteria for IBS were enrolled [8,14]. Organic diseases were ruled out through detailed clinical history, physical examination, and negative auxiliary examination. Further details are provided in the Supplementary Methods (Supplementary File 2). 49 patients were excluded due to severe missing questionnaire data that could not be reasonably imputed to meet the requirements of statistical analysis. Ultimately, 419 patients were included in the analysis. Based on fulfilment of the Rome III and Rome IV diagnostic criteria, all included patients were classified into three diagnostic categories: Rome III only, Rome IV only, and both Rome III and Rome IV. This classification was used to describe the overlap between the two diagnostic criteria and the distribution of patients across different diagnostic categories. In subsequent analyses, patients were further categorized according to Rome IV diagnostic status into Rome IV criteria-eligible and Rome IV criteria-ineligible groups to evaluate the association between Rome IV classification and disease burden. The enrolment, exclusion, and grouping process is shown in Figure 1.

### Data collection and assessment tools

All enrolled IBS patients underwent face-to-face interviews conducted by experienced clinicians with expertise in the diagnosis and management of IBS. Detailed information was collected, including demographic characteristics, overlapping upper gastrointestinal symptoms, IBS symptoms, somatization symptoms, anxiety and depressive symptoms, and quality of life. Demographic characteristics included gender, age, body mass index (BMI), marital status, education level, occupation, smoking status, and alcohol status.

Overlapping upper gastrointestinal symptoms were defined as the presence of one or more upper gastrointestinal symptoms occurring at least once per week during the past three months in patients with IBS. These symptoms included acid regurgitation, heartburn, belching, globus sensation, dysphagia, retrosternal pain, anorexia, food regurgitation, rumination, nausea, vomiting, early satiety, postprandial fullness, and epigastric pain [15]. Abdominal pain, bloating, and discomfort were considered IBS-related abdominal symptoms, and were not included in the definition of overlapping upper gastrointestinal symptoms. The proportion of days with IBS-related abdominal symptoms was calculated as the number of days with relevant symptoms during the past 100 days divided by the total number of days (100 days). IBS-related defecatory symptoms included urgent, difficult, and incomplete defecation.

Stool consistency was evaluated using the Bristol Stool Form Scale (BSFS) (Figure S1) [16]. The severity of IBS-related abdominal and defecatory symptoms was assessed using a patient-reported scoring system. IBS symptom severity was evaluated using the Irritable Bowel Syndrome Severity Scoring System (IBS-SSS) [11]. Somatization symptoms were assessed using the 12-item Patient Health Questionnaire (PHQ-12), while anxiety and depressive symptoms were evaluated using the Self-Rating Anxiety Scale (SAS) and Self-Rating Depression Scale (SDS), respectively [12,17,18]. Health-related quality of life was assessed using the 36-Item Short Form Health Survey (SF-36) [19–21]. Detailed criteria can be found in the Supplementary File 2.

### LCA of IBS burden phenotypes

LCA is a clustering approach based on finite mixture models and probability theory [22]. It identifies unobserved heterogeneity among individuals by examining patterns of observed variables and classifies individuals with similar characteristics into distinct latent classes. This approach enables detailed phenotypic stratification of study populations. Compared with conventional clustering methods, LCA does not require predefined assumptions about data distributions or subjective distance measures. It can simultaneously handle different types of variables and determine the optimal number of classes using model fit indices, including the log-likelihood, Akaike information criterion (AIC), and Bayesian information criterion (BIC). In addition, LCA estimates the posterior probability of class membership for each individual, improving the statistical robustness and clinical interpretability of the identified phenotypes. LCA has been widely applied in gastrointestinal disorders and psychiatric conditions to identify disease heterogeneity, define clinical phenotypes, and explore personalized treatment strategies [10,23].

The poLCA package in R software was used to construct latent class models with 2–6 classes, using the severity of IBS symptoms, somatization symptoms, and anxiety and depression symptoms as observed variables in this study. Health-related quality of life was used as an independent disease burden indicator to validate differences between latent classes. The optimal number of classes was determined based on model fit indices, clinical interpretability, and model parsimony.

### Machine learning-based construction of the IBS-BI

Least absolute shrinkage and selection operator (LASSO) regression and random forest regression were jointly applied to develop the IBS-BI. LASSO regression is a linear regression model based on L1 regularization [24]. By introducing a penalty term, LASSO shrinks some coefficients to zero, enabling feature selection and reducing the risk of overfitting. Random forest regression is an ensemble machine learning method based on multiple decision trees [25]. It can capture potential nonlinear relationships and complex interactions among variables. As IBS disease burden involves multiple dimensions, including gastrointestinal symptoms, psychological status, and quality of life, complex nonlinear associations may exist among these factors. Therefore, random forest regression was selected over traditional linear regression to construct a composite disease burden index.

The IBS-BI was developed based on multidimensional assessments from the IBS-SSS, PHQ-12, SAS, SDS, and SF-36. SF-36 scores were reverse-coded. A higher positivized SF-36 value indicates a worse outcome. All candidate variables were then standardized using Z-score transformation to eliminate differences in measurement scales. Subsequently, LASSO regression was performed using the glmnet package in R to select key features for model development. The study population was randomly divided into a training set and an internal validation set at a 7:3 ratio. A random forest regression model was developed based on the features selected by LASSO regression using the randomForest package in R (ntree = 500). The variable importance was calculated using the built-in importance() function from the randomForest package. The feature importance score was based on the increase in node purity (IncNodePurity), which reflects the contribution of each variable to improving node purity across all trees in the random forest model. The relative weight proportion of each feature was subsequently calculated by normalizing the IncNodePurity values according to the following formula: *Relative contribution (%) = IncNodePurity_i_/∑IncNodePurity_i_×100%*. The model-predicted values were used as the initial IBS-BI scores. These scores were then normalized using min-max scaling to generate standardized IBS-BI scores ranging from 0 to 1. Model performance was evaluated using the coefficient of determination (R^2^), mean squared error (MSE), and mean absolute error (MAE). Only internal validation was performed in this study, and no independent external cohort validation was conducted.

Based on the distribution of standardized IBS-BI scores in the study population, the 33rd (P33) and 66th (P66) percentiles were used as cut-off values to classify patients into mild, moderate, and severe IBS-BI severity groups. Since no validation was performed using independent cohorts, these severity subgroups are regarded as preliminary statistical classifications. In addition, an interactive clinical calculator was developed using the Shiny package in R to enable individualized calculation of IBS-BI scores.

### Statistical analysis

All statistical analyses were conducted using R software (Version 4.5.3, R Core Team, R Foundation for Statistical Computing, Vienna, Austria), with results validated using IBM SPSS Statistics 26.0 (IBM Corp., Armonk, NY, USA).

Continuous variables were assessed for normality and homogeneity of variance. Normally distributed variables with equal variances were presented as mean ± standard deviation (SD). Comparisons between two groups were performed using the independent-samples Student’s t-test, while comparisons among multiple groups were performed using one-way analysis of variance (ANOVA) followed by Tukey’s honestly significant difference (HSD) test for post-hoc pairwise comparisons. For normally distributed variables with unequal variances, Welch’s correction was applied. Non-normally distributed continuous variables were presented as median (P25, P75). Comparisons between two groups were performed using the Mann-Whitney U test, and comparisons among multiple groups were performed using the Kruskal-Wallis H test followed by Dunn (Bonferroni) test for post-hoc pairwise comparisons. Categorical variables were expressed as numbers and percentages (n, %). Chi-square tests were used for comparisons of nominal categorical variables. Ordinal categorical variables were compared using the Mann-Whitney U test for two groups and the Kruskal-Wallis H test for multiple groups. All statistical tests were two-sided, and *p* <0.05 was considered statistically significant.

## Results

### Baseline characteristics and diagnostic classification of IBS patients

Among the 468 patients initially diagnosed with IBS according to the Rome III and/or Rome IV criteria, 49 patients were excluded due to substantial missing questionnaire data. Finally, 419 patients were included in the analysis. Based on the original diagnostic classifications, patients were categorized into three diagnostic categories: Rome III only (*n* = 199), Rome IV only (*n* = 2), and both Rome III and Rome IV (*n* = 218). Only two patients met the Rome IV criteria but not the Rome III criteria, whereas most patients meeting the Rome IV criteria also met the Rome III criteria. The demographic and clinical characteristics of these diagnostic categories are presented in Table 1. Statistical comparisons were not performed because the Rome IV-only category included only two patients.

**Table 1.** Distribution and baseline characteristics of IBS patients according to Rome III and Rome IV diagnostic classifications.

Characteristics	Rome III only (*n* = 199)	Rome IV only (*n* = 2)	Both Rome III and IV (*n* = 218)
Gender, n (%)			
Male	104 (52.26)	1 (50.00)	135 (61.93)
Female	95 (47.74)	1 (50.00)	83 (38.07)
Average age, years	44.73	58.00	42.49
Average BMI, kg/m²	23.79	23.40	23.68
Marital status, n (%)			
Unmarried	28 (14.07)	0 (0.00)	44 (20.18)
Married	159 (79.90)	2 (100.00)	161 (73.85)
Divorced/Widowed	12 (6.03)	0 (0.00)	13 (5.96)
Education level, n (%)			
Primary school or below	30 (15.08)	0 (0.00)	19 (8.72)
Junior high school	40 (20.10)	1 (50.00)	39 (17.89)
Senior high school	37 (18.59)	0 (0.00)	32 (14.68)
Undergraduate	80 (40.20)	1 (50.00)	102 (46.79)
Postgraduate	12 (6.03)	0 (0.00)	26 (11.93)
Occupation, n (%)			
Manual	38 (19.10)	1 (50.00)	38 (17.43)
Non-manual	133 (66.83)	0 (0.00)	156 (71.56)
Not working	28 (14.07)	1 (50.00)	24 (11.01)
Smoking status, n (%)	67 (33.67)	0 (0.00)	54 (24.77)
Alcohol status, n (%)	49 (24.62)	0 (0.00)	42 (19.27)
IBS subtype, n (%)			
IBS-C	31 (15.58)	0 (0.00)	17 (7.80)
IBS-D	135 (67.84)	2 (100.00)	174 (79.82)
IBS-M	8 (4.02)	0 (0.00)	8 (3.67)
IBS-U	25 (12.56)	0 (0.00)	19 (8.72)

Abbreviations: BMI, body mass index; IBS, irritable bowel syndrome; IBS-C, IBS with predominant constipation; IBS-D, IBS with predominant diarrhoea; IBS-M, IBS with mixed bowel habits; IBS-U, IBS unclassified.

For subsequent analyses, patients were further categorized according to Rome IV diagnostic status into Rome IV criteria-eligible (*n* = 220) and Rome IV criteria-ineligible (*n* = 199) groups. The corresponding demographic and disease characteristics are shown in Table S1.

### Comparison of overlapping upper gastrointestinal symptoms according to Rome IV diagnostic status

Regarding overlapping upper gastrointestinal symptoms, anorexia was less common in the Rome IV criteria-eligible group than in the Rome IV criteria-ineligible group (10.00% vs 34.67%, *p* <0.001) (Table S2). No significant differences were observed between the two groups for the remaining individual upper gastrointestinal symptoms and the total number of overlapping upper gastrointestinal symptoms (all *p* > 0.05).

### Comparison of IBS symptoms according to Rome IV diagnostic status

**Figure 2.**
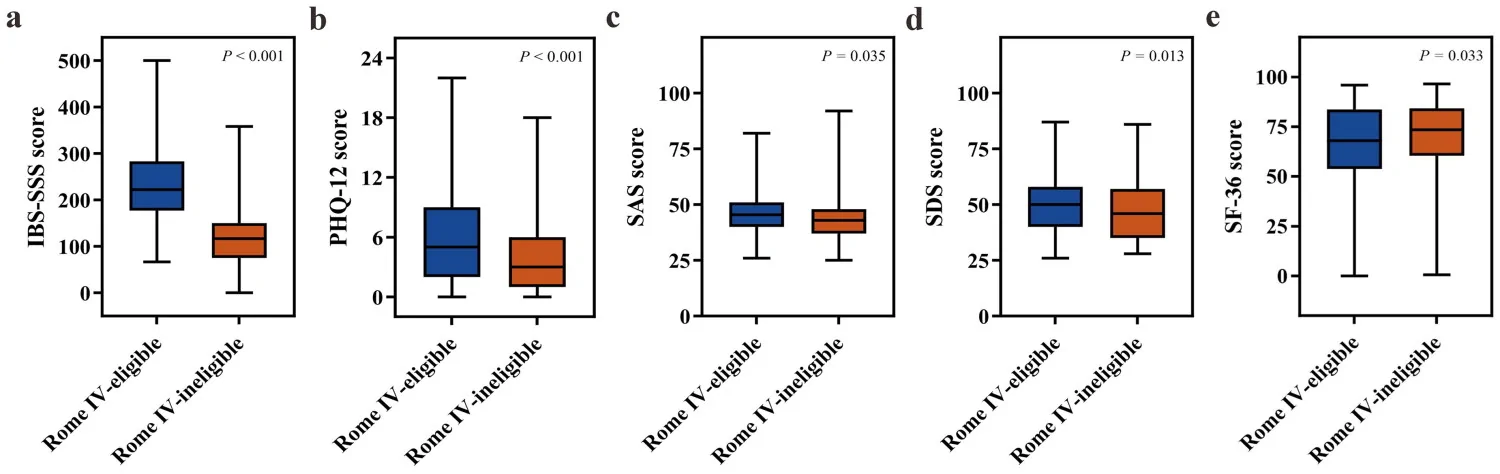
Comparison of IBS symptoms, somatization symptoms, anxiety symptoms, depression symptoms, and health-related quality of life according to Rome IV diagnostic status among IBS patients. (a) IBS-SSS score; (b) PHQ-12 score; (c) SAS score; (d) SDS score; (e) SF-36 score. IBS, irritable bowel syndrome; IBS-SSS, IBS-Severity Scoring System; PHQ-12, Patient Health Questionnaire-12; SAS, Self-Rating Anxiety Scale; SDS, Self-Rating Depression Scale; SF-36, 36-Item Short Form Health Survey.

The IBS-SSS score was significantly higher in the Rome IV criteria-eligible group than in the Rome IV criteria-ineligible group [222.03 (177.38, 283.34) vs 116.66 (75.00, 150.00), *p* <0.001] (Figure 2a; Table 2). According to IBS-SSS severity categories, only one patient (0.45%) in the Rome IV criteria-eligible group had very mild or almost normal symptoms, whereas 50 (22.73%), 125 (56.82%), and 44 (20.00%) patients had mild, moderate, and severe symptoms, respectively. In contrast, 60 (30.15%), 113 (56.78%), 24 (12.06%), and 2 (1.01%) patients in the Rome IV criteria-ineligible group had very mild or almost normal symptoms, mild symptoms, moderate symptoms, and severe symptoms, respectively. The distribution of IBS symptom severity differed significantly between the two groups (*p* <0.001).

**Table 2.** Comparison of IBS symptoms, somatization symptoms, anxiety symptoms, depression symptoms according to Rome IV diagnostic status among IBS patients.

Characteristics	Rome IV criteria-eligible group (*n* = 220)	Rome IV criteria-ineligible group (*n* = 199)	*P* value
IBS-SSS score [Median (P25, P75)]	222.03 (177.38, 283.34)	116.66 (75.00, 150.00)	<0.001^a^
IBS symptoms, n (%)			
Very mild/Almost normal	1 (0.45)	60 (30.15)	<0.001^a^
Mild	50 (22.73)	113 (56.78)	
Moderate	125 (56.82)	24 (12.06)	
Severe	44 (20.00)	2 (1.01)	
PHQ-12 score [Median (P25, P75)]	5.00 (2.00, 9.00)	3.00 (1.00, 6.00)	<0.001^a^
Somatization symptoms, n (%)			
None	84 (38.18)	101 (50.75)	0.001^a^
Mild	65 (29.55)	66 (33.17)	
Moderate	51 (23.18)	20 (10.05)	
Severe	20 (9.09)	12 (6.03)	
SAS score [Median (P25, P75)]	45.50 (40.00, 51.00)	43.00 (37.00, 48.00)	0.035^a^
Anxiety symptoms, n (%)			
None	146 (66.36)	150 (75.38)	0.061^a^
Mild	59 (26.82)	36 (18.09)	
Moderate	13 (5.91)	10 (5.03)	
Severe	2 (0.91)	3 (1.51)	
SDS score [Median (P25, P75)]	50.00 (40.00, 58.00)	46.00 (35.00, 57.00)	0.013^a^
Depression symptoms, n (%)			
None	124 (56.36)	136 (68.34)	0.002^a^
Mild	63 (28.64)	42 (21.11)	
Moderate	27 (12.27)	17 (8.54)	
Severe	6 (2.73)	4 (2.01)	

^a^Mann-Whitney U test.

Abbreviations: IBS, irritable bowel syndrome; IBS-SSS, IBS-Severity Scoring System; PHQ-12, Patient Health Questionnaire-12; SAS, Self-Rating Anxiety Scale; SDS, Self-Rating Depression Scale.

Specifically, the comparison of IBS-related abdominal symptoms is presented in Table S3. Significant differences were observed between the Rome IV criteria-eligible and Rome IV criteria-ineligible groups in abdominal pain (*p* <0.001) and abdominal discomfort (*p* <0.001). All patients in the Rome IV criteria-eligible group reported abdominal pain, whereas only 22 patients (11.06%) in the Rome IV criteria-ineligible group reported this symptom. In contrast, abdominal discomfort was more common in the Rome IV criteria-ineligible group than in the Rome IV criteria-eligible group (71.86% vs 10.00%). No significant difference was found in bloating between the two groups (*p* = 0.139). In addition, the proportion of days with IBS-related abdominal symptoms was significantly higher in the Rome IV criteria-eligible group than in the Rome IV criteria-ineligible group (60.00% vs 43.00%, *p* <0.001). IBS-related defecatory symptoms are summarized in Table S4. Compared with the Rome IV criteria-ineligible group, patients in the Rome IV criteria-eligible group more frequently reported severe urgent defecation (*p* <0.001) and incomplete defecation (*p* = 0.008). No significant difference was observed in difficult defecation between the two groups (*p* = 0.668).

### Comparison of somatization, anxiety, and depression symptoms according to Rome IV diagnostic status

As for somatization symptoms, the median PHQ-12 score was significantly higher in the Rome IV criteria-eligible group than in the Rome IV criteria-ineligible group [5.00 (2.00, 9.00) vs 3.00 (1.00, 6.00), *p* <0.001] (Figure 2b; Table 2). Based on PHQ-12 severity categories, 84 patients (38.18%) in the Rome IV criteria-eligible group had no somatization symptoms, while 65 (29.55%), 51 (23.18%), and 20 (9.09%) patients had mild, moderate, and severe symptoms, respectively. In comparison, the Rome IV criteria-ineligible group had a higher proportion of patients without somatization symptoms (50.75%, *n* = 101), with a significant difference in symptom distribution between the two groups (*p* = 0.001).

The median SAS and SDS scores were also significantly higher in the Rome IV criteria-eligible group than in the Rome IV criteria-ineligible group [SAS: 45.50 (40.00, 51.00) vs 43.00 (37.00, 48.00), *p* = 0.035; SDS: 50.00 (40.00, 58.00) vs 46.00 (35.00, 57.00), *p* = 0.013] (Figure 2c, 2d; Table 2). Stratified analysis based on SDS severity showed a significant difference in depression symptom distribution between the two groups (*p* = 0.002). Although anxiety severity showed a similar trend, no statistically significant difference was observed based on SAS classification (*p* = 0.061).

### Comparison of health-related quality of life according to Rome IV diagnostic status

The median SF-36 score was significantly lower in the Rome IV criteria-eligible group than in the Rome IV criteria-ineligible group [68.05 (56.90, 78.10) vs 73.50 (62.90, 82.90), *p* = 0.033] (Figure 2e; Table 3). The median physical component summary score was significantly lower in the Rome IV criteria-eligible group than in the Rome IV criteria-ineligible group (*p* = 0.001), whereas no significant difference was observed in the median mental component summary score (70.84 vs 72.92, *p* = 0.525). Among the eight SF-36 domains, the Rome IV criteria-eligible group had significantly lower scores for physical functioning (*p* <0.001) and bodily pain (*p* <0.001). No significant differences were found in role physical, general health, vitality, social functioning, role emotional, or mental health between the two groups (all *p* > 0.05) (Figure S2).

**Table 3.** Comparison of health-related quality of life according to Rome IV diagnostic status among IBS patients.

Characteristics	Rome IV criteria-eligible group (*n* = 220)	Rome IV criteria-ineligible group (*n* = 199)	*P* value
SF-36 score [Median (P25, P75)]	68.05 (53.82, 83.63)	73.50 (60.42, 84.25)	0.033^a^
Physical component summary [Median (P25, P75)]	69.50 (51.56, 81.25)	75.50 (64.75, 84.75)	0.001^a^
Physical functioning [Median (P25, P75)]	95.00 (85.00, 100.00)	100.00 (90.00, 100.00)	<0.001^a^
Role physical [Median (P25, P75)]	75.00 (25.00, 100.00)	100.00 (50.00, 100.00)	0.105^a^
Bodily pain [Median (P25, P75)]	66.00 (52.50, 84.00)	84.00 (64.00, 100.00)	<0.001^a^
General health [Median (P25, P75)]	45.00 (30.00, 59.25)	47.00 (35.00, 62.00)	0.105^a^
Mental component summary [Median (P25, P75)]	70.84 (53.55, 86.00)	72.92 (52.96, 86.75)	0.525^a^
Vitality [Median (P25, P75)]	65.00 (50.00, 78.75)	65.00 (55.00, 80.00)	0.449^a^
Social functioning [Median (P25, P75)]	87.50 (62.50, 100.00)	75.00 (62.50, 100.00)	0.865^a^
Role emotional [Median (P25, P75)]	100.00 (33.33, 100.00)	100.00 (33.33, 100.00)	0.403^a^
Mental health [Median (P25, P75)]	64.00 (52.00, 80.00)	64.00 (48.00, 76.00)	0.445^a^

^a^Mann-Whitney U test.

Abbreviations: IBS, irritable bowel syndrome; SF-36, 36-Item Short Form Health Survey.

### Independent factors associated with health-related quality of life in IBS patients

As shown in Table 4, the multivariable linear regression model was statistically significant (*F* = 19.342, *p* <0.001), with an adjusted R^2^ of 0.401, explaining 40.1% of the variance in SF-36 scores. The Durbin-Watson statistic was 1.979, and all variance inflation factor values were below 10, indicating no significant autocorrelation or severe multicollinearity. Higher IBS-SSS (*p* <0.001), PHQ-12 (*p* <0.001), SAS (*p* = 0.002), and SDS (*p* <0.001) scores were independently associated with lower SF-36 scores. Alcohol consumption was independently associated with higher SF-36 scores (*p* = 0.014). No other evaluated factors showed significant associations with health-related quality of life (all *p* > 0.05).

**Table 4.** Multivariable linear regression analysis of factors associated with health-related quality of life in IBS patients.

Characteristics	Unstandardized β	95% CI for β	Standardized β	t value	*P* value	VIF
(Constant)	114.996	100.245 to 129.746	–	15.326	<0.001	–
Gender						
Male	Reference					
Female	−0.278	−3.458 to 2.902	−0.008	−0.172	0.864	1.322
Age	−0.062	−0.194 to 0.069	−0.047	−0.931	0.352	1.736
BMI	−0.063	−0.456 to 0.331	−0.013	−0.313	0.755	1.095
Marital status						
Unmarried	Reference					
Married	3.107	−1.180 to 7.394	0.072	1.425	0.155	1.747
Divorced/Widowed	6.321	−1.211 to 13.853	0.083	1.650	0.100	1.701
Education level	0.471	−0.981 to 1.922	0.031	0.638	0.524	1.599
Occupation						
Not working	Reference					
Manual	−3.638	−9.066 to 1.791	−0.078	−1.317	0.188	2.362
Non-manual	−4.483	−9.000 to 0.035	−0.115	−1.951	0.052	2.333
Smoking status	−2.634	−6.207 to 0.938	−0.066	−1.449	0.148	1.401
Alcohol status	4.749	0.974 to 8.524	0.108	2.473	0.014	1.294
IBS-SSS score	−0.033	−0.050 to −0.016	−0.163	−3.783	<0.001	1.259
PHQ-12 score	−0.998	−1.404 to −0.591	−0.240	−4.826	<0.001	1.671
SAS score	−0.339	−0.549 to −0.130	−0.178	−3.182	0.002	2.115
SDS score	−0.339	−0.488 to −0.189	−0.237	−4.449	<0.001	1.913

Abbreviations: BMI, body mass index; CI, confidence interval; IBS, irritable bowel syndrome; IBS-SSS, IBS-Severity Scoring System; PHQ-12, Patient Health Questionnaire-12; SAS, Self-Rating Anxiety Scale; SDS, Self-Rating Depression Scale; VIF, variance inflation factor.

### Distinct IBS burden phenotypes identified by LCA

Based on the differences observed between the Rome IV criteria-eligible and Rome IV criteria-ineligible groups and the results of multivariable linear regression, LCA was performed using IBS symptoms, somatization symptoms, anxiety symptoms, and depression symptoms as indicator variables. Health-related quality of life was used as a validation measure to assess differences among latent classes. Models containing 2–6 latent classes were constructed, and model fit was evaluated using the log-likelihood, AIC, and BIC (Table S5). Considering model fit, clinical interpretability, and parsimony, the four-class model was selected as the optimal solution.

**Figure 3.**
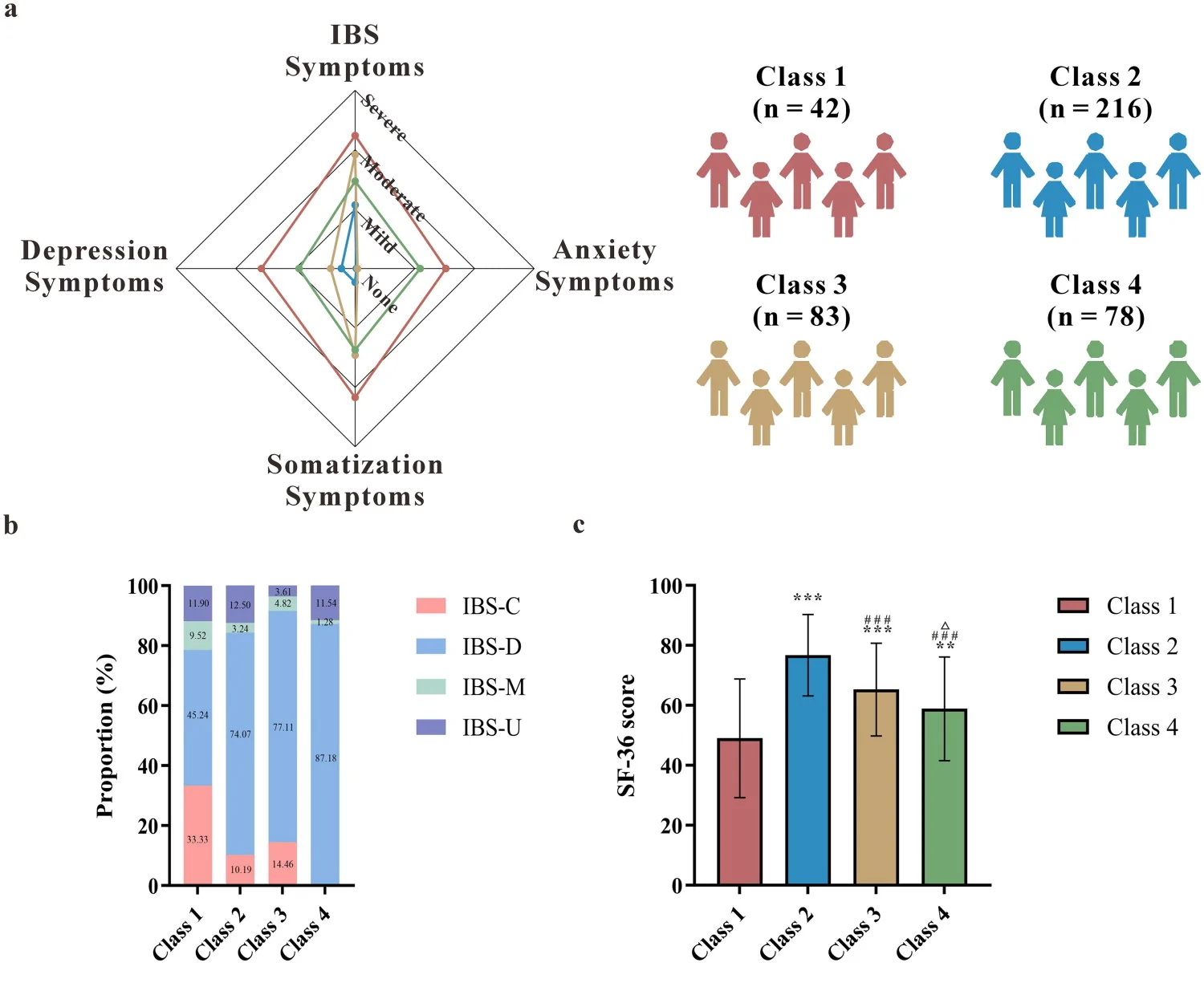
Symptom characteristics, IBS subtype distribution, and health-related quality of life across latent classes identified by LCA. (a) Radar chart showing multidimensional symptom characteristics of the four latent classes, including IBS symptoms, somatization symptoms, anxiety symptoms, and depression symptoms. (b) Distribution of Rome criteria-defined IBS subtypes across the four latent classes. (c) Comparison of SF-36 scores among the four latent classes (higher scores indicate better health-related quality of life). ***p <0.001 vs Class 1, ^###^*p* <0.001 vs Class 2, ^##^*p* <0.01 vs Class 2, ^△^ *p* <0.05 vs Class 3. IBS, irritable bowel syndrome; IBS-C, IBS with constipation; IBS-D, IBS with diarrhoea; IBS-M, mixed IBS; IBS-U, unsubtyped IBS; LCA, latent class analysis; SF-36, 36-Item Short Form Health Survey.

The characteristics of the four latent classes are presented in Figure 3a and Table 5. Class 1 was identified as the high-symptom burden phenotype, characterized by the highest levels of IBS, somatization, anxiety, and depression symptoms, together with the poorest health-related quality of life. Traditional IBS subtypes were widely distributed in this phenotype, with higher proportions of IBS-C, IBS-D, IBS-M, and IBS-U compared with other classes (Figure 3b). Class 2 represented the low-symptom burden phenotype, characterized by relatively low symptom severity across all domains and the highest health-related quality of life among the four classes. IBS-D was the predominant traditional subtype in this class, whereas other subtypes were uncommon. Class 3 was characterized as the somatization-dominant phenotype, with moderate IBS symptoms, prominent somatization symptoms, relatively low anxiety and depression symptoms, and intermediate health-related quality of life. IBS-D was also the most common subtype in this phenotype. Class 4 was identified as the psychological comorbidity phenotype, characterized by mild IBS symptoms, moderate somatization symptoms, and marked anxiety and depression symptoms, accompanied by reduced health-related quality of life. Most patients in this phenotype had IBS-D, while IBS-C was not observed.

**Table 5.** Characteristics of the four latent class-derived IBS burden phenotypes.

Characteristics	^a^Class 1 (*n* = 42)	^b^Class 2 (*n* = 216)	^c^Class 3 (*n* = 83)	^d^Class 4 (*n* = 78)
Class proportions, %	10.02	51.55	19.81	18.62
Severity of IBS symptoms, Mean ± SD	3.24 ± 0.53	2.07 ± 0.74	2.92 ± 0.67	2.47 ± 0.99
Severity of somatization symptoms, Mean ± SD	3.17 ± 1.06	1.23 ± 0.48	2.46 ± 0.59	2.37 ± 0.77
Severity of anxiety symptoms, Mean ± SD	2.52 ± 0.67	1.02 ± 0.19	1.04 ± 0.33	2.09 ± 0.29
Severity of depression symptoms, Mean ± SD	2.57 ± 0.91	1.23 ± 0.55	1.41 ± 0.63	1.94 ± 0.74
Health-related quality of life, Mean ± SD	49.01 ± 19.80	76.73 ± 13.57	65.28 ± 15.45	58.84 ± 17.29
IBS subtype, n (%)				
IBS-C	14 (33.33)	22 (10.19)	12 (14.46)	0 (0.00)
IBS-D	19 (45.24)	160 (74.07)	64 (77.11)	68 (87.18)
IBS-M	4 (9.52)	7 (3.24)	4 (4.82)	1 (1.28)
IBS-U	5 (11.90)	27 (12.50)	3 (3.61)	9 (11.54)

^a^Class 1, high-symptom burden phenotype.

^b^Class 2, low-symptom burden phenotype.

^c^Class 3, somatization-dominant phenotype.

^d^Class 4, psychological comorbidity phenotype.

Abbreviations: IBS, irritable bowel syndrome; IBS-C, IBS with predominant constipation; IBS-D, IBS with predominant diarrhoea; IBS-M, IBS with mixed bowel habits; IBS-U, IBS unclassified; SD, standard deviation.

Significant differences in health-related quality of life were observed among the four phenotypes (*p* <0.05), with post-hoc comparisons showing significant differences between all class pairs (all *p* <0.05) (Figure 3c; Table S6). In addition, the four phenotypes differed significantly in IBS symptoms, somatization symptoms, anxiety symptoms, and depression symptoms (all *p* <0.001).

### Development and evaluation of the IBS-BI and its clinical calculator

The IBS-BI was developed based on IBS symptoms, somatization symptoms, anxiety symptoms, depression symptoms, and health-related quality of life. The optimal penalty coefficient for the LASSO model was λ = 0.07946 after 10-fold cross-validation (Figure S3). IBS-SSS, PHQ-12, SAS, SDS, and positivized SF-36 standardized scores were retained as the five core features for IBS-BI construction (Table S7).

The study population was randomly divided into a training set (*n* = 295, 70.41%) and an internal validation set (*n* = 124, 29.59%) at a 7:3 ratio. No significant differences were observed in core scale scores or baseline characteristics between the two sets (all *p* > 0.05), indicating good baseline comparability (Table S8).

**Figure 4.**
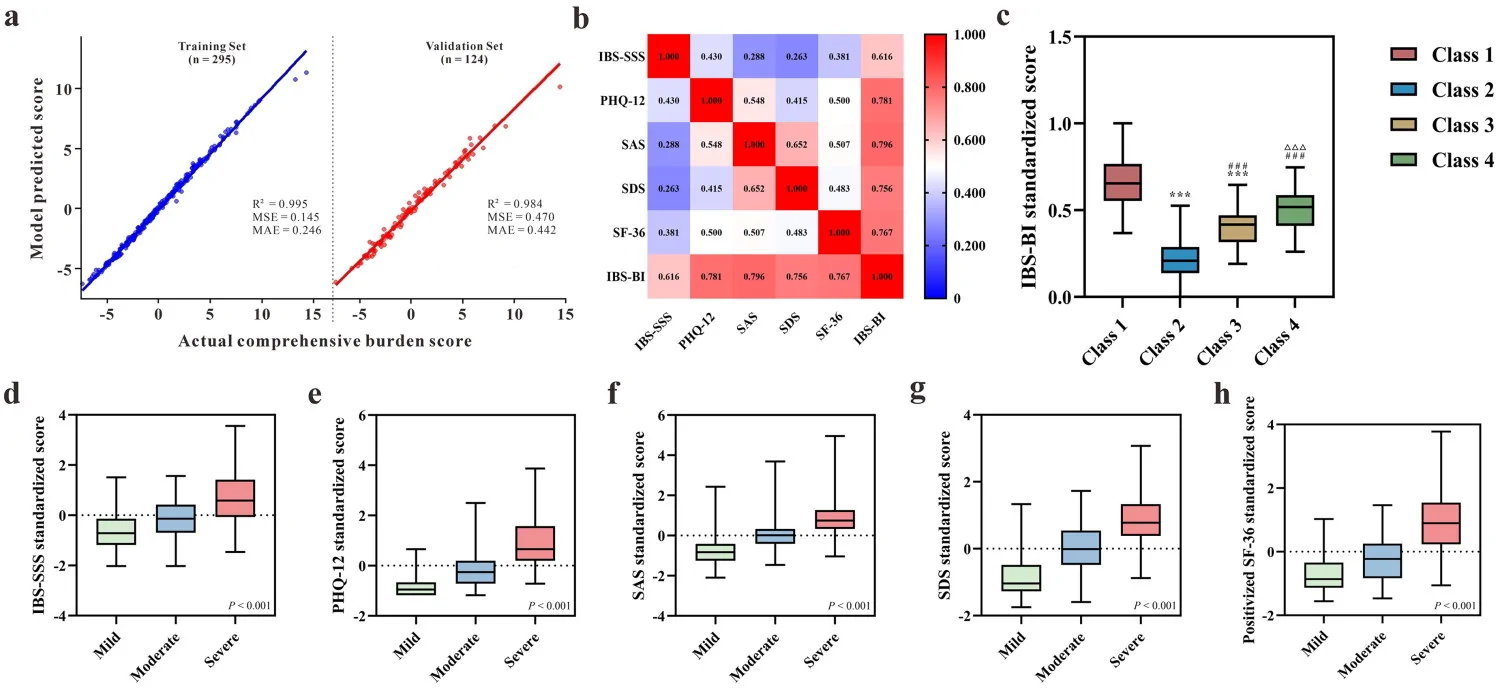
Construction and validation of the IBS-BI and its ability to characterize disease burden. (a) Model performance validation in the 7:3 randomly split training set (left) and validation set (right), with predicted versus actual comprehensive burden scores. (b) Correlation matrix showing associations between IBS-BI and its core features. (c) Comparison of IBS-BI standardized scores among four latent classes derived from LCA. (d–h) Comparison of IBS symptoms, somatization symptoms, anxiety symptoms, depression symptoms, and health-related quality of life across mild, moderate, and severe IBS-BI groups. ***p <0.001 vs Class 1, ^###^*p* <0.001 vs Class 2, ^△△△^*p* <0.001 vs Class 3. IBS, irritable bowel syndrome; IBS-BI, IBS Burden Index; IBS-SSS, IBS-Severity Scoring System; LCA, latent class analysis; MAE, mean absolute error; MSE, mean squared error; PHQ-12, Patient Health Questionnaire-12; SAS, Self-Rating Anxiety Scale; SDS, Self-Rating Depression Scale; SF-36, 36-Item Short Form Health Survey.

A random forest regression model was subsequently developed using the five selected features. Feature importance analysis demonstrated different contributions of individual variables to model performance. Based on IncNodePurity, the positivized SF-36 score showed the highest importance, whereas PHQ-12 contributed most to predictive accuracy according to percentage increase in MSE (%IncMSE) (Figure S3). The relative contribution weights calculated from IncNodePurity ranked the five features as follows: positivized SF-36 (22.5%), SAS (20.8%), PHQ-12 (20.6%), SDS (20.1%), and IBS-SSS (16.0%) (Table 6). Model performance evaluation showed that the random forest model achieved R^2^, MSE, and MAE values of 0.995, 0.145, and 0.246 in the training set, and 0.984, 0.470, and 0.442 in the internal validation set, respectively, indicating good model fit and internal stability (Figure 4a). Pearson correlation analysis revealed significant positive correlations between IBS-BI and all core features (all *p* <0.001), with the strongest correlations observed for SAS (*r* = 0.796) and PHQ-12 (*r* = 0.781), followed by positivized SF-36 (*r* = 0.767), SDS (*r* = 0.756), and IBS-SSS (*r* = 0.616) (Figure 4b). Further analysis demonstrated significant variation in IBS-BI standardized scores across the four LCA-derived phenotypes (*p* <0.001) (Figure 4c; Table S9). Post-hoc testing showed that most phenotype pairs differed significantly, except for Class 1 versus Class 4 (*p* = 0.113). Specifically, significant differences were observed between Class 1 and Class 2, Class 1 and Class 3, Class 2 and Class 3, Class 2 and Class 4 (all *p* <0.001), and Class 3 and Class 4 (*p* = 0.009).

**Table 6.** Feature importance and relative contribution of variables in the random forest regression model.

Characteristics	Feature importance score	Weight proportion (%)
IBS-SSS standardized score	650.799	16.0
PHQ-12 standardized score	837.704	20.6
SAS standardized score	847.755	20.8
SDS standardized score	820.636	20.1
Positivized SF-36 standardized score	918.840	22.5

Abbreviations: IBS, irritable bowel syndrome; IBS-SSS, IBS-Severity Scoring System; PHQ-12, Patient Health Questionnaire-12; SAS, Self-Rating Anxiety Scale; SDS, Self-Rating Depression Scale; 36-Item Short Form Health Survey.

Based on the distribution of IBS-BI standardized scores, the P33 and P66 cut-off values were 0.233 and 0.422, respectively, classifying the 419 patients into mild (< 0.233) (*n* = 138, 32.94%), moderate (≥ 0.233 and < 0.422) (*n* = 138, 32.94%), and severe (≥ 0.422) (*n* = 143, 34.13%) severity groups, which were interpreted as preliminary statistical classifications until validated in independent cohorts. The distribution of patients across the three groups was comparable. Significant differences were observed among the three groups in IBS-SSS, PHQ-12, SAS, SDS, and positivized SF-36 standardized scores (all *p* <0.001), with increasing IBS-BI levels associated with greater IBS symptoms, somatization symptoms, anxiety symptoms and depression symptoms, and poorer health-related quality of life (Figure 4d–h). Post-hoc analyses confirmed significant differences between all pairwise comparisons (all *p* <0.001) (Table S10).

Furthermore, an interactive clinical calculator was developed using the R Shiny package (https://wyb000926.shinyapps.io/ibs_bi_web/), strictly adhering to the programming environment standards used in the model construction, ensuring that the development environment is fully consistent with the modeling environment. All core model parameters and indicator transformation rules were pre-locked, ensuring the stability, consistency, and reproducibility of the output results from a technical perspective, thus meeting the reproducibility requirements for clinical research. This calculator incorporates the final random forest regression model established in this study and can completely reproduce the process from inputting raw scale scores to outputting the IBS-BI standardized score, automating and standardizing both data processing and result output. The interface design adopts a user-friendly dual-column layout (Figure S4). The left input module clearly labels the valid scoring ranges for each scale (IBS-SSS, PHQ-12, SAS, SDS, SF-36), effectively preventing invalid inputs and improving operational convenience. The right output module provides real-time feedback on the IBS-BI standardized score and the corresponding disease burden stratification results after clicking the Calculate button. This calculator requires no installation of specialized statistical software and can be accessed directly *via* mainstream web browsers, compatible with multiple devices such as computers and tablets.

## Discussion

In this multicentre cross-sectional study, we systematically compared IBS patients who met and did not meet the Rome IV diagnostic criteria in terms of demographic characteristics, overlapping upper gastrointestinal symptoms, IBS symptoms, somatization symptoms, anxiety symptoms, depression symptoms, and health-related quality of life. Based on multidimensional clinical features, we identified four distinct IBS phenotypes with different clinical characteristics. Furthermore, we developed and internally validated the IBS-BI as a quantitative tool for assessing overall disease burden in IBS patients and established an interactive web-based calculator to facilitate its potential application in clinical assessment and future research. However, as IBS-BI has currently undergone only internal validation, its generalizability and clinical utility require further confirmation in independent external cohorts.

This study found that patients fulfilling the Rome IV criteria had greater symptom burden, more prominent psychological symptoms, and poorer health-related quality of life, which is consistent with previous studies [26–29]. However, these findings should be interpreted in the context of the diagnostic characteristics of the Rome IV criteria. The Rome IV criteria require the presence of recurrent abdominal pain associated with defecation, occurring at least once per week during the previous 3 months [8,14,30]. The observed differences in disease burden may also largely reflect the higher symptom threshold of the Rome IV criteria rather than indicating an inherent superiority of Rome IV criteria in identifying patients with greater disease burden. In addition, patients meeting the Rome IV criteria showed several demographic differences, including a higher proportion of males, higher educational attainment, and lower smoking rates. These findings are partly consistent with previous studies [31,32]. Such differences may be related to the stricter symptom requirements of the Rome IV criteria. The higher diagnostic threshold may preferentially identify individuals with greater symptom awareness, higher health literacy, and a stronger tendency to seek medical care. The proportion of IBS-D was higher among patients fulfilling the Rome IV criteria than among those who did not (80.00% vs 67.84%). This finding is consistent with a previous multinational study conducted by Oka et al. involving 34 countries [33]. One possible explanation is that patients with IBS-D may have a stronger association between abdominal pain and bowel movements, making them more likely to meet the core diagnostic requirement of the Rome IV criteria.

Meanwhile, multivariable linear regression analysis further demonstrated that greater severity of IBS symptoms, somatization symptoms, anxiety, and depression were independently associated with poorer health-related quality of life. This finding is in line with the study by Trindade et al. using structural equation modeling, further supporting the concept that IBS is a multidimensional disorder involving gastrointestinal symptoms, somatic symptoms, and psychological factors [34]. In clinical practice, management strategies should not only focus on improving gastrointestinal symptoms but also address somatization symptoms and psychological comorbidities. Notably, alcohol-consuming patients showed better health-related quality of life in this study. However, this finding should be interpreted with caution. In this study, alcohol use was assessed only as a binary variable (yes/no), without further evaluation of consumption amount, frequency, or drinking patterns. Therefore, the observed association does not indicate a causal relationship or a beneficial effect of alcohol consumption. In addition, alcohol use may be influenced by potential confounding factors, including social participation, occupational status, age, and disease severity. For example, patients with milder symptoms and better social functioning may be more likely to participate in social activities involving alcohol consumption, which could partially explain this association. Further studies with detailed assessment of alcohol exposure and comprehensive adjustment for potential confounders are needed to clarify this relationship.

Based on the previous study by Han et al. which identified IBS subtypes using LCA, we further incorporated somatization symptoms and anxiety/depression-related features to capture a broader spectrum of clinical characteristics [9]. Four clinically distinct IBS phenotypes were identified: high-symptom burden phenotype, low-symptom burden phenotype, somatization-dominant phenotype, and psychological comorbidity phenotype. Compared with the seven-category classification proposed by Black et al. in 2023, the major contribution of our study was the inclusion of somatization features [10]. Cluster analysis has become one of the most widely used approaches for identifying novel IBS classifications and phenotypes. Both k-means clustering and LCA have been frequently applied in previous studies. To date, more than 10 studies have used clustering methods to define IBS subtypes [35]. Despite differences in classification strategies and selected variables, most studies have consistently shown that a large proportion of IBS patients have relatively mild symptom severity and better quality of life, which is also consistent with our findings.

The recently proposed Rome V criteria further emphasize that IBS is a biopsychosocial disorder influenced by gastrointestinal symptoms, somatization symptoms, psychological factors, and health-related quality of life [36]. Therefore, comprehensive management strategies may be required for IBS patients. However, individual indicators usually reflect only specific aspects of the disease and cannot fully capture the overall disease burden. For example, some patients may have high IBS-SSS scores but mild psychological symptoms and limited impairment in quality of life. In contrast, other patients may present with moderate gastrointestinal symptoms accompanied by significant anxiety, depression, and reduced quality of life. Relying solely on symptom severity may underestimate the overall burden and management needs of the latter group. In clinical practice, IBS patients often undergo multiple assessments, including IBS-SSS, PHQ-12, SAS, SDS, and SF-36. However, these scales provide fragmented information, and the overall assessment of disease status may largely depend on clinicians’ experience, introducing potential subjectivity. By integrating multiple clinical dimensions, IBS-BI may provide a unified measure of disease burden and may facilitate the identification of patients with different levels of overall disease impact. Furthermore, IBS-BI was developed using a random forest algorithm, which allows the model to capture potential complex interactions among clinical features rather than simply combining individual scores. By providing an integrated measure, IBS-BI may help evaluate changes in overall disease burden and provide a reference for clinical research, outcome evaluation, and comparisons of different intervention strategies. For instance, a decrease in IBS-BI score from 0.72 to 0.35 after treatment may indicate an improvement in the overall disease burden. Importantly, IBS-BI was not developed to replace existing individual assessment tools, but rather to complement them. The composite index provides an overall evaluation of disease burden, while specific scales can further identify the major contributing factors. To facilitate the accessibility and practical application of IBS-BI, we further developed an interactive clinical calculator based on the R Shiny platform. This tool enables rapid calculation of IBS-BI scores and corresponding burden categories using commonly available clinical scale scores. It may provide a convenient approach for comprehensive assessment of IBS burden in clinical and research settings.

This study has several limitations. Although identical standardized diagnostic procedures, questionnaires, interviewer training, and data collection protocols were applied at both centres, the long interval between patient recruitment periods may have introduced temporal heterogeneity, including changes in clinical practice, implementation of the Rome IV criteria, treatment strategies, and the potential impact of the COVID-19 pandemic. Another limitation is that the IBS-BI model was developed and internally validated within the current cohort, without external validation. Although the model demonstrated strong internal performance, its generalizability to other populations remains uncertain. Future studies should validate the IBS-BI model in independent external cohorts from different regions and clinical settings, and further assess its calibration and clinical applicability before widespread implementation. Furthermore, the lack of longitudinal follow-up prevented evaluation of the analyses of treatment outcomes, therapeutic algorithms, or clinical utility. Finally, this study only collected information on whether patients consumed alcohol and did not assess the consumption amount, frequency, or drinking patterns. Therefore, the observed association between alcohol consumption and quality of life should be interpreted cautiously, and further studies with more detailed assessments of alcohol exposure are needed.

## Conclusions

Patients with IBS who fulfilled the Rome IV criteria showed a higher disease burden, which may be related to the more stringent diagnostic requirements of these criteria. Based on multidimensional clinical characteristics, this study further identified distinct IBS phenotypes and developed the IBS-BI together with an accompanying online calculator, providing a novel methodological approach for comprehensive assessment of IBS. The IBS-BI may have potential value for evaluating disease burden and patient stratification. However, its clinical utility and broader applicability require further validation in independent external cohorts and prospective studies.

## Supplementary Material

Supplementary File 2.docx

Supplementary File 1.docx

## Data Availability

The datasets generated and/or analysed during the current study are not publicly available due to but are available from the corresponding author on reasonable request.
