# Supplementary material for: Development and internal validation of a machine learning-based disease burden index for irritable bowel syndrome: a multicentre cross-sectional study
Source: Ann Med. 2026 Aug 20;58(1):2721087. doi: 10.1080/07853890.2026.2721087 (PMC13495042; doi:10.1080/07853890.2026.2721087)
Supplement: Supplementary file 2 [file IANN_A_2721087_SM1851.docx]

**Catalogue**

[1. Supplementary Methods	1](#_Toc28102)

[2. Supplementary Figures	6](#_Toc7381)

[3. Supplementary Tables	11](#_Toc21062)

1. **Supplementary Methods**

***Auxiliary examination and exclusion criteria for IBS***

The auxiliary examination included complete blood count, C-reactive protein, routine thyroid function tests, stool analysis (white blood cell count, red blood cell count, and fecal occult blood test), colonoscopy, and abdominal ultrasonography. Gastroscopy was additionally performed in patients with IBS who had upper gastrointestinal symptoms or were older than 40 years to rule out organic upper gastrointestinal diseases.

Exclusion criteria were as follows: (1) presence of gastrointestinal alarm features, such as hematochezia, positive fecal occult blood test, anemia, abdominal mass, ascites, fever, or unexplained weight loss; (2) concomitant organic gastrointestinal disease, including inflammatory bowel disease, intestinal tuberculosis, gastrointestinal tumors, or intestinal obstruction, as well as a history of gastrointestinal surgery or cholecystectomy; (3) a prior confirmed diagnosis of extra-gastrointestinal organic disease, such as tuberculous peritonitis, cholelithiasis, liver cirrhosis, or chronic pancreatitis; (4) underlying conditions that may affect gastrointestinal function, including hyperthyroidism or hypothyroidism, diabetes mellitus, endometriosis, chronic renal insufficiency, autoimmune diseases, and neurological disorders such as Parkinson’s disease; (5) severe cardiac, pulmonary, cerebral, renal, hematological, or other major organ diseases, as well as malignant tumors; (6) IBS-like symptoms induced by medication use.

***Rome III Diagnostic Criteria for IBS***

Symptoms must have occurred for at least 6 months prior to diagnosis, with the following criteria met in the last 3 months: recurrent abdominal pain or discomfort, occurring at least 3 days per month in the past 3 months, accompanied by two or more of the following: (1) relief of symptoms after defecation; (2) changes in stool frequency during episodes; (3) changes in stool consistency (appearance) during episodes.

The following symptoms support the diagnosis of IBS: (1) abnormal stool frequency (fewer than three bowel movements per week or more than three bowel movements per day); (2) abnormal stool consistency (hard, lumpy stools or loose, watery stools); (3) straining during defecation; (4) urgency or incomplete evacuation during defecation; (5) mucus in stools; (6) bloating.

***Rome IV Diagnostic Criteria for IBS***

Symptoms must have occurred for at least 6 months prior to diagnosis, with the following criteria met in the last 3 months: recurrent abdominal pain, occurring on average at least 1 day per week in the past 3 months, accompanied by two or more of the following: (1) abdominal pain related to defecation; (2) changes in stool frequency during episodes; (3) changes in stool consistency (appearance) during episodes.

The following symptoms support the diagnosis of IBS: (1) abnormal stool frequency (fewer than three bowel movements per week or more than three bowel movements per day); (2) abnormal stool consistency (hard, lumpy stools or loose, watery stools); (3) straining during defecation; (4) urgency or incomplete evacuation during defecation; (5) mucus in stools; (6) bloating.

***Rome III and Rome IV Subtyping Criteria for IBS***

Rome III subtyping criteria for IBS: (1) IBS-C is defined as having > 25% of hard or lumpy stools (BSFS Type 1-2) and < 25% of loose or watery stools (BSFS Type 6-7); (2) IBS-D is defined as having > 25% of loose or watery stools (BSFS Type 6-7) and < 25% of hard or lumpy stools (BSFS Type 1-2); (3) IBS-M is defined as having > 25% of both hard or lumpy stools (BSFS Type 1-2) and loose or watery stools (BSFS Type 6-7); (4) IBS-U is defined as meeting the IBS Rome III diagnostic criteria but having abnormal stool characteristics that do not fulfill the criteria for any of the three above types.

Rome IV subtyping criteria for IBS: (1) IBS-C is defined as having ≥ 25% of hard or lumpy stools (BSFS Type 1-2) and < 25% of loose or watery stools (BSFS Type 6-7); (2) IBS-D is defined as having ≥ 25% of loose or watery stools (BSFS Type 6-7) and < 25% of hard or lumpy stools (BSFS Type 1-2); (3) IBS-M is defined as having ≥ 25% of both hard or lumpy stools (BSFS Type 1-2) and loose or watery stools (BSFS Type 6-7); (4) IBS-U is defined as meeting the IBS Rome IV diagnostic criteria but having both abnormal proportions of hard or lumpy stools (BSFS Type 1-2) and loose or watery stools (BSFS Type 6-7) of < 25%, and cannot be classified into any of the three types above.

***Patient-reported scoring system***

The patient-reported scoring system ranged from 1 to 100 points. Patients completed the assessment independently, without being informed of the specific severity categories. Based on the scores, symptom severity was classified as mild (1-33 points), moderate (34-66 points), or severe (67-100 points).

***IBS-SSS***

IBS-SSS is an effective tool for assessing the severity of IBS symptoms, which is mainly used in clinical practice and research to monitor treatment efficacy over time, and assist in clinical decision-making. The scale consists of 5 items, which assess the intensity of abdominal pain, the frequency of abdominal pain episodes, the severity of bloating, satisfaction with bowel movements, and the impact of gastrointestinal symptoms on daily life. Each item is graded based on the severity of symptoms, with a total score ranging from 0 to 500. The severity grading criteria are as follows: mild (75-175 points), moderate (176-300 points), severe (> 300 points), and < 75 points is considered as very mild or almost normal IBS symptoms.

***PHQ-12***

PHQ-12 is a simplified revision of the PHQ-15 somatic symptom scale, which removes three gastrointestinal-related items (nausea, diarrhea, and constipation) to focus on assessing non-gastrointestinal somatic symptoms. This approach effectively avoids interference from primary gastrointestinal symptoms, making it suitable for evaluating somatic symptoms in patients with functional gastrointestinal disorders, such as IBS. The scale includes 12 self-reported items, divided into four assessment dimensions. The pain dimension covers back pain, limb pain, headache, chest pain, dysmenorrhea, and dyspareunia (6 items), the autonomic symptoms dimension includes dizziness, fainting, palpitations, and shortness of breath (4 items), while the remaining 2 items assess fatigue and sleep disturbances. The scale uses a 0-2 point grading system, where 0 indicates no distress, 1 indicates mild distress, and 2 indicates severe distress. The total score ranges from 0 to 24 points, with a higher score indicating more severe somatization. The corresponding severity levels are as follows: ≤ 3 points for no or mild somatization, 4-7 points for mild somatization, 8-12 points for moderate somatization, and ≥ 13 points for severe somatization.

***SAS***

SAS was developed by Zung in 1971 and consists of 20 self-reported items, covering dimensions such as mental, somatic anxiety, and motor restlessness. The scale has good reliability and validity and is suitable for self-assessment and monitoring of anxiety symptoms in adults. The scale uses a 1-4 grading system, and the raw score is multiplied by 1.25 to obtain the standard score. The higher the score, the more severe the anxiety. The standard score interpretation is as follows: < 50 points indicates normal, 50-59 points indicates mild anxiety, 60-69 points indicates moderate anxiety, and ≥ 70 points indicates severe anxiety.

***SDS***

SDS, also developed by Zung, consists of 20 self-reported items used to assess the depressive mood over the past week, covering emotional, somatic, and psychological dimensions. The scale uses a 1-4 grading system, and the raw score is multiplied by 1.25 to obtain the standard total score. According to Chinese norms, the standard score interpretation is as follows: < 53 points indicates no depression, 53-62 points indicates mild depression, 63-72 points indicates moderate depression, and ≥ 73 points indicates severe depression. A higher score indicates more severe depressive symptoms.

***SF-36***

The quality of life was assessed using the SF-36, a scale developed by Ware et al. in 1992 based on medical outcome research, with a Chinese version revised and domestic norms established by Professor Fang Jiqian’s team. The SF-36 is suitable for assessing the quality of life and intervention effects in populations with functional gastrointestinal disorders and other chronic diseases. The scale includes 36 self-reported items, covering eight core dimensions: physical functioning, role physical, bodily pain, general health, vitality, social functioning, role emotional, and mental health. These can be summarized into physical component summary and mental component summary. The raw scores of each dimension are converted to a scale of 0-100, with higher scores indicating better health-related quality of life.

2. **Supplementary Figures**

**Figure S1**. Bristol Stool Form Scale.

**Figure S2.** Comparison of health-related quality of life according to Rome IV diagnostic status among IBS patients

**Figure S3.** Feature selection and importance evaluation for the construction of the IBS-BI

**Figure S4.** User interface of the interactive IBS-BI clinical calculator

**Figure S1.** BSFS. *BSFS, Bristol Stool Form Scale.*

**Figure S2.** Comparison of health-related quality of life according to Rome IV diagnostic status among IBS patients. (a) Physical component summary; (b) Mental component summary; (c) Physical functioning; (d) Role physical; (e) Vitality; (f) Social functioning; (g) Bodily pain; (h) General health; (i) Role emotional; (j) Mental health. *IBS, irritable bowel syndrome; BP, bodily pain; GH, general health; MCS, mental component summary; MH, mental health; PCS, physical component summary; PF, physical functioning; RE, role emotional; RP, role physical; SF, social functioning; SF-36, 36-Item Short Form Health Survey; VT, vitality.*

**Figure S3.** Feature selection and importance evaluation for the construction of the IBS-BI. (a) Selection of the optimal penalty parameter (λ) in the LASSO regression model using 10-fold cross-validation. (b) Variable importance based on the percentage increase in MSE (%IncMSE) from the random forest model. (c) Variable importance based on the IncNodePurity from the random forest model. *IBS, irritable bowel syndrome; IBS-BI, IBS burden index; IBS-SSS, IBS-Severity Scoring System; LASSO, least absolute shrinkage and selection operator; MSE, mean squared error; PHQ-12, Patient Health Questionnaire-12; SAS, Self-Rating Anxiety Scale; SDS, Self-Rating Depression Scale; SF-36, 36-Item Short Form Health Survey.*

**Figure S4.** User interface of the interactive IBS-BI clinical calculator. *IBS, irritable bowel syndrome; IBS-BI, IBS Burden Index; IBS-SSS, IBS-Severity Scoring System; PHQ-12, Patient Health Questionnaire-12; SAS, Self-Rating Anxiety Scale; SDS, Self-Rating Depression Scale; SF-36, 36-Item Short Form Health Survey.*

3. **Supplementary Tables**

**Table S1.** Demographic and disease characteristics of IBS patients according to Rome IV diagnostic status

**Table S2.** Comparison of overlapping upper gastrointestinal symptoms according to Rome IV diagnostic status among IBS patients

**Table S3.** Comparison of IBS-related abdominal symptoms according to Rome IV diagnostic status among IBS patients

**Table S4.** Comparison of IBS-related defecatory symptoms according to Rome IV diagnostic status among IBS patients

**Table S5.** Model fit indices for latent class models with 2-6 classes

**Table S6.** Comparison of symptom severity and health-related quality of life across four IBS burden phenotypes

**Table S7.** LASSO regression coefficients for feature selection in IBS-BI construction

**Table S8.** Comparison of baseline characteristics between the training and internal validation sets

**Table S9.** Comparison of IBS-BI standardized scores across four latent classes identified by LCA

**Table S10.** Comparison of core characteristics among different IBS-BI groups with post-hoc analyses

| **Table S1.** Demographic and disease characteristics of IBS patients according to Rome IV diagnostic status | | | |
| --- | --- | --- | --- |
| **Characteristics** | **Rome IV criteria-eligible group**  **(n = 220)** | **Rome IV criteria-ineligible group**  **(n = 199)** | ***P* value** |
| Gender, n (%) |  |  |  |
| Male | 136 (61.82) | 104 (52.26) | 0.048^a^ |
| Female | 84 (38.18) | 95 (47.74) |  |
| Age [Median (P25, P75), years] | 40.00 (31.00, 54.75) | 43.00 (34.00, 55.00) | 0.050^b^ |
| BMI [Median (P25, P75), kg/m²] | 23.66 (21.27, 25.85) | 23.44 (21.22, 25.71) | 0.972^b^ |
| Marital status, n (%) |  |  |  |
| Unmarried | 44 (20.00) | 28 (14.07) | 0.288^a^ |
| Married | 163 (74.09) | 159 (79.90) |  |
| Divorced/Widowed | 13 (5.91) | 12 (6.03) |  |
| Education level, n (%) |  |  |  |
| Primary school or below | 19 (8.64) | 30 (15.08) | 0.004^b^ |
| Junior high school | 40 (18.18) | 40 (20.10) |  |
| Senior high school | 32 (14.55) | 37 (18.59) |  |
| Undergraduate | 103 (46.82) | 80 (40.20) |  |
| Postgraduate | 26 (11.82) | 12 (6.03) |  |
| Occupation, n (%) |  |  |  |
| Manual | 39 (17.73) | 38 (19.10) | 0.606^a^ |
| Non-manual | 156 (70.91) | 133 (66.84) |  |
| Not working | 25 (11.36) | 28 (14.07) |  |
| Smoking status, n (%) | 54 (24.55) | 67 (33.67) | 0.040^a^ |
| Alcohol status, n (%) | 42 (19.09) | 49 (24.62) | 0.170^a^ |
| IBS subtype, n (%) |  |  |  |
| IBS-C | 17 (7.73) | 31 (15.58) | 0.026^a^ |
| IBS-D | 176 (80.00) | 135 (67.84) |  |
| IBS-M | 8 (3.64) | 8 (4.02) |  |
| IBS-U | 19 (8.64) | 25 (12.56) |  |
| ^a^Chi-square test, ^b^Mann-Whitney U test. | | | |
| *Abbreviations: BMI, body mass index; IBS, irritable bowel syndrome; IBS-C, IBS with predominant constipation; IBS-D, IBS with predominant diarrhea; IBS-M, IBS with mixed bowel habits; IBS-U, IBS unclassified.* | | | |

| **Table S2.** Comparison of overlapping upper gastrointestinal symptoms according to Rome IV diagnostic status among IBS patients | | | |
| --- | --- | --- | --- |
| **Symptoms** | **Rome IV criteria-eligible group**  **(n = 220)** | **Rome IV criteria-ineligible group**  **(n = 199)** | ***P* value** |
| Acid regurgitation, n (%) | 56 (25.45) | 45 (22.61) | 0.497^a^ |
| Heartburn, n (%) | 50 (22.73) | 48 (24.12) | 0.737^a^ |
| Belching, n (%) | 67 (30.45) | 75 (37.69) | 0.118^a^ |
| Globus sensation, n (%) | 14 (6.36) | 13 (6.53) | 0.944^a^ |
| Dysphagia, n (%) | 7 (3.18) | 3 (1.51) | 0.423^a^ |
| Retrosternal pain, n (%) | 16 (7.27) | 17 (8.54) | 0.630^a^ |
| Anorexia, n (%) | 22 (10.00) | 69 (34.67) | < 0.001^a^ |
| Food regurgitation, n (%) | 13 (5.91) | 17 (8.54) | 0.296^a^ |
| Rumination, n (%) | 4 (1.82) | 4 (2.01) | 1.000^a^ |
| Nausea, n (%) | 26 (11.82) | 16 (8.04) | 0.198^a^ |
| Vomiting, n (%) | 1 (0.45) | 6 (3.01) | 0.097^a^ |
| Early satiety, n (%) | 26 (11.82) | 29 (14.57) | 0.404^a^ |
| Postprandial fullness, n (%) | 61 (27.73) | 61 (30.65) | 0.510^a^ |
| Epigastric pain, n (%) | 39 (17.73) | 28 (14.07) | 0.308^a^ |
| Total number of overlapping upper gastrointestinal symptoms  [Median (P25, P75)] | 1 (0, 3) | 1 (1, 3) | 0.092^b^ |
| ^a^Chi-square test, ^b^Mann-Whitney U test. | | | |
| *Abbreviations: IBS, irritable bowel syndrome.* | | | |

| **Table S3.** Comparison of IBS-related abdominal symptoms according to Rome IV diagnostic status among IBS patients | | | |
| --- | --- | --- | --- |
| **Symptoms** | **Rome IV criteria-eligible group**  **(n = 220)** | **Rome IV criteria-ineligible group**  **(n = 199)** | ***P* value** |
| Abdominal pain, n (%) |  |  |  |
| None | 0 (0.00) | 177 (88.94) | < 0.001^a^ |
| Mild | 53 (24.09) | 11 (5.53) |  |
| Moderate | 108 (49.09) | 7 (3.52) |  |
| Severe | 59 (26.82) | 4 (2.01) |  |
| Abdominal bloating, n (%) |  |  |  |
| None | 112 (50.91) | 85 (42.71) | 0.139^a^ |
| Mild | 37 (16.82) | 36 (18.09) |  |
| Moderate | 32 (14.55) | 42 (21.11) |  |
| Severe | 39 (17.73) | 36 (18.09) |  |
| Abdominal discomfort, n (%) |  |  |  |
| None | 198 (90.00) | 56 (28.14) | < 0.001^a^ |
| Mild | 7 (3.18) | 72 (36.18) |  |
| Moderate | 11 (5.00) | 50 (25.13) |  |
| Severe | 4 (1.82) | 21 (10.55) |  |
| Proportion of days with IBS-related abdominal symptoms  [Median (P25, P75), %] | 60.00 (33.00, 100.00) | 43.00 (10.00, 100.00) | < 0.001^a^ |
| ^a^Mann-Whitney U test. | | | |
| *Abbreviations: IBS, irritable bowel syndrome.* | | | |

| **Table S4.** Comparison of IBS-related defecatory symptoms according to Rome IV diagnostic status among IBS patients | | | |
| --- | --- | --- | --- |
| **Symptoms** | **Rome IV criteria-eligible group**  **(n = 220)** | **Rome IV criteria-ineligible group**  **(n = 199)** | ***P* value** |
| Urgent defecation, n (%) |  |  |  |
| None | 55 (25.00) | 76 (38.19) | < 0.001^a^ |
| Mild | 112 (50.91) | 102 (51.26) |  |
| Moderate | 24 (10.91) | 12 (6.03) |  |
| Severe | 29 (13.18) | 9 (4.52) |  |
| Difficult defecation, n (%) |  |  |  |
| None | 154 (70.00) | 141 (70.85) | 0.668^a^ |
| Mild | 47 (21.36) | 48 (24.12) |  |
| Moderate | 8 (3.64) | 6 (3.02) |  |
| Severe | 11 (5.00) | 4 (2.01) |  |
| Incomplete defecation, n (%) |  |  |  |
| None | 73 (33.18) | 86 (43.22) | 0.008^a^ |
| Mild | 102 (46.36) | 90 (45.23) |  |
| Moderate | 28 (12.73) | 14 (7.04) |  |
| Severe | 17 (7.73) | 9 (4.52) |  |
| ^a^Mann-Whitney U test. | | | |
| *Abbreviations: IBS, irritable bowel syndrome.* | | | |

| **Table S5.** Model fit indices for latent class models with 2-6 classes | | | |
| --- | --- | --- | --- |
| **Number of Classes** | **Log-likelihood** | **AIC** | **BIC** |
| 2 | -1997.86 | 4057.73 | 4182.90 |
| 3 | -1981.13 | 4056.26 | 4246.04 |
| 4 | -1964.40 | 4054.81 | 4309.19 |
| 5 | -1955.50 | 4069.00 | 4387.99 |
| 6 | -1941.86 | 4073.73 | 4457.33 |
| *Abbreviations: AIC, Akaike information criterion; BIC, Bayesian information criterion.* | | | |

| **Table S6.** Comparison of symptom severity and health-related quality of life across four IBS burden phenotypes | | | | |
| --- | --- | --- | --- | --- |
| **Characteristics** | **Statistical Test** | **Test Statistic** | ***P* value** | **Post hoc Comparisons**  **(Tukey’s HSD, 95% CI)** |
| Severity of IBS symptoms | Kruskal-Wallis H test | χ² = 104.81, df = 3 | < 0.001 | Not applicable |
| Severity of somatization symptoms | Kruskal-Wallis H test | χ² = 239.51, df = 3 | < 0.001 | Not applicable |
| Severity of anxiety symptoms | Kruskal-Wallis H test | χ² = 389.89, df = 3 | < 0.001 | Not applicable |
| Severity of depression symptoms | Kruskal-Wallis H test | χ² = 130.35, df = 3 | < 0.001 | Not applicable |
| Health-related quality of life | One-way ANOVA | *F*_(3, 415)_ = 54.49 | < 0.001 | Class 2 vs Class 1:  diff = 27.72 (21.02, 34.41)  *P* < 0.001  Class 3 vs Class 1:  diff = 16.27 (8.76, 23.79)  *P* < 0.001  Class 4 vs Class 1:  diff = 9.83 (2.24, 17.43)  *P* = 0.005  Class 3 vs Class 2:  diff = -11.45 (-16.57, -6.32)  *P* < 0.001  Class 4 vs Class 2:  diff = -17.88 (-23.13, -12.64)  *P* < 0.001  Class 4 vs Class 3:  diff = -6.44 (-12.70, -0.18)  *P* = 0.041 |
| *Abbreviations: ANOVA, analysis of variance; CI, confidence interval; HSD, honestly significant difference; IBS, irritable bowel syndrome.* | | | | |

| **Table S7.** LASSO regression coefficients for feature selection in IBS-BI construction | |
| --- | --- |
| **Characteristics** | **Standardized coefficients (β)** |
| IBS-SSS standardized score | 0.954 |
| PHQ-12 standardized score | 0.977 |
| SAS standardized score | 0.981 |
| SDS standardized score | 0.967 |
| Positivized SF-36 standardized score | 0.975 |
| *Abbreviations: IBS, irritable bowel syndrome; IBS-BI, IBS Burden Index; IBS-SSS, IBS-Severity Scoring System; LASSO, least absolute shrinkage and selection operator; PHQ-12, Patient Health Questionnaire-12; SAS, Self-Rating Anxiety Scale; SDS, Self-Rating Depression Scale; SF-36, 36-Item Short Form Health Survey.* | |

| **Table S8.** Comparison of baseline characteristics between the training and internal validation sets | | | |
| --- | --- | --- | --- |
| **Characteristics** | **Training set**  **(n = 295)** | **Validation set**  **(n = 124)** | ***P* value** |
| IBS-SSS score, Median (P25, P75) | 175.00 (116.67, 235.71) | 170.24 (108.33, 243.46) | 0.998^a^ |
| PHQ-12 score, Median (P25, P75) | 4.00 (2.00, 7.00) | 5.00 (2.00, 8.00) | 0.452^a^ |
| SAS score, Median (P25, P75) | 45.00 (38.00, 51.00) | 44.00 (40.00, 50.00) | 0.942^a^ |
| SDS score, Median (P25, P75) | 48.00 (37.00, 57.00) | 47.50 (38.00, 58.00) | 0.792^a^ |
| SF-36 score, Median (P25, P75) | 71.21 (56.19, 83.88) | 71.78 (57.63, 83.98) | 0.689^a^ |
| ^a^Mann-Whitney U test. | | | |
| *Abbreviations: IBS, irritable bowel syndrome; IBS-SSS, IBS-Severity Scoring System; PHQ-12, Patient Health Questionnaire-12; SAS, Self-Rating Anxiety Scale; SDS, Self-Rating Depression Scale; SF-36, 36-Item Short Form Health Survey.* | | | |

| **Table S9.** Comparison of IBS-BI standardized scores across four latent classes identified by LCA | | | | | |
| --- | --- | --- | --- | --- | --- |
| **Characteristics** | **Statistical Test** | **Test Statistic** | ***P* value** | **Post-hoc test** | ***P* value** |
| IBS-BI standardized score | Kruskal-Wallis | 263.67 | < 0.001 | Dunn (Bonferroni) | Class 1 vs Class 2:  *P* < 0.001  Class 1 vs Class 3:  *P* < 0.001  Class 1 vs Class 4:  *P* = 0.113  Class 2 vs Class 3:  *P* < 0.001  Class 2 vs Class 4:  *P* < 0.001  Class 3 vs Class 4:  *P* = 0.009 |
| *Abbreviations: IBS, irritable bowel syndrome; IBS-BI, IBS Burden Index; LCA, latent class analysis.* | | | | | |

| **Table S10.** Comparison of core characteristics among different IBS-BI groups with post-hoc analyses | | | | | |
| --- | --- | --- | --- | --- | --- |
| **Characteristics** | **Statistical Test** | **Test Statistic** | ***P* value** | **Post-hoc test** | ***P* value** |
| IBS-SSS standardized score | Kruskal-Wallis | 122.545 | < 0.001 | Dunn (Bonferroni) | Mild vs Moderate:  *P* < 0.001  Mild vs Severe:  *P* < 0.001  Moderate vs Severe:  *P* < 0.001 |
| PHQ-12 standardized score | Kruskal-Wallis | 235.913 | < 0.001 | Dunn (Bonferroni) | Mild vs Moderate:  *P* < 0.001  Mild vs Severe:  *P* < 0.001  Moderate vs Severe:  *P* < 0.001 |
| SAS standardized score | Kruskal-Wallis | 222.874 | < 0.001 | Dunn (Bonferroni) | Mild vs Moderate:  *P* < 0.001  Mild vs Severe:  *P* < 0.001  Moderate vs Severe:  *P* < 0.001 |
| SDS standardized score | One-way ANOVA | 202.067 | < 0.001 | Tukey’s HSD | Mild vs Moderate:  *P* < 0.001  Mild vs Severe:  *P* < 0.001  Moderate vs Severe:  *P* < 0.001 |
| Positivized SF-36 standardized score | Kruskal-Wallis | 190.251 | < 0.001 | Dunn (Bonferroni) | Mild vs Moderate:  *P* < 0.001  Mild vs Severe:  *P* < 0.001  Moderate vs Severe:  *P* < 0.001 |
| *Abbreviations: ANOVA, analysis of variance; HSD, honestly significant difference; IBS, irritable bowel syndrome; IBS-BI, IBS Burden Index; IBS-SSS, IBS-Severity Scoring System; PHQ-12, Patient Health Questionnaire-12; SAS, Self-Rating Anxiety Scale; SDS, Self-Rating Depression Scale; SF-36, 36-Item Short Form Health Survey.* | | | | | |
